# Bombay Blood Group and Pregnancy

**DOI:** 10.18502/ijph.v49i7.3593

**Published:** 2020-07

**Authors:** Jing QIAN, Hong-Bo ZHAI

**Affiliations:** Department of Obsterics, Hangzhou First People’s Hospital, Zhejiang University School of Medicine, Hangzhou, Zhejiang 310006, China

## Dear Editor-in-Chief

Bombay blood type was known as the rarest in the world. The probability of finding a person with Bombay was one for every 250.000 worldwide (1). Bombay individuals was characterized by the absence of A, B and H antigens on red blood cells and associated with anti-H antibodies in plasma. The anti-H antibodies in the maternal blood could cause hemolytic disease in fetus and may trigger severe transfusion reaction especially hemolysis when exposed to any blood type except Bombay. Scattered cases reported the managements of pregnant women with Bombay blood type.

Here, we introduce a rare case of twin pregnancy in a women with Bombay blood group. A 32-year-old woman, at 35 gestation weeks, was hospitalized for regular uterine contraction in Apr 2017. The women was diagnosed with Bombay blood type and twin pregnancy. Cesarean delivery was decided because of breech presentation. Connection with the central blood bank was made immediately to assure the corresponding blood. The twins were successfully delivered with normal Apgar score. However, the placenta adhered tightly to uterine wall. Manual stripping of the placenta was quickly implemented. The uterus was still soft and poorly contracted after placenta ablation and bleeding profusely. Uterine massage was then performed and uterotonic agents was administered sequentially to strengthen uterine contraction. After the incision was sutured, the uterine contraction was still weak.

Then surgical technique (the B-lynch procedure) was conducted to control the bleeding. Then the bleeding gradually stopped. The cumulative blood loss during the operation was estimated 1000ML. Two units of red blood cell was transfused to increase the blood volume and maintained hemodynamic stability.

Two articles showed the Bombay’s immune system was extremely sensitive to even small amount of group O or other group donar cells (2,3). Thus irregular antibody screening was advised in pregnant women to distinguish Bombay in time. Maternal hemorrhage was the leading cause of maternal mortality worldwide. The blood product should be in preparation in case of the need of transfusion. Because of the rarity of the Bombay blood and limited donors, autologous blood donation was conducted to collect packed red blood cells. The safety of autologous blood donation during pregnancy was reported in some articles (4,5). Moreover, every obstetric members should master the process procedure of postpartum hemorrhage and prepare to manage woman who experience it.

The maternal production of immunoglobulin G anti-H during pregnancy could cause hemolytic disease in fetus who did not inherit the mother’s Bombay phenotype. Monitoring the neonate for developing jaundice was needed for the activity of IgG anti-H. Evaluation of middle cerebral artery peak systolic velocity through ultrasound was suggested to monitor the potential fetal hemolytic disease and anemia resulting from maternal anti-H IgG antibodies.

We hope that the obstericians pay more attention to the perinatal health care of the women with Bombay blood type.
